# Mechanism of Endoplasmic Reticulum Stress Pathway in the Osteogenic Phenotypic Transformation of Aortic Valve Interstitial Cells

**DOI:** 10.3389/fendo.2022.856331

**Published:** 2022-03-08

**Authors:** Yiming Tao, Yimin Geng, Wenpei Dang, Xinxin Xu, Hui Zhao, Lijuan Zou, Yongsheng Li

**Affiliations:** ^1^ Department of Intensive Care Medicine, Tongji Hospital, Tongji Medical College, Huazhong University of Science and Technology, Wuhan, China; ^2^ The Emergency Department, Tongji Hospital, Tongji Medical College, Huazhong University of Science and Technology, Wuhan, China

**Keywords:** aortic valve calcification, endoplasmic reticulum stress, valve interstitial cells, ox-LDL, TUDCA, BMP2

## Abstract

**Background and Purpose:**

Calcific Aortic Valve Disease (CAVD) is a crucial component of degenerative valvular disease in old age and with the increasing prevalence of the aging population. we hope that by modeling valvular osteogenesis and intervening with endoplasmic reticulum stress inhibitor TUDCA to observe the effect of endoplasmic reticulum stress on valve osteogenesis

**Methods:**

In this study, rabbit heart valvular interstitial cells (VICs) were isolated and cultured. They treated with ox-LDL (Oxidized Low Density Lipoprotein) stimulation to establish a model of valvular osteogenic transformation. BMP2 (Bone Morphogenetic Protein 2), PERK (Protein kinase R-like endoplasmic reticulum kinase), CHOP (CCAAT/enhancer-binding protein homologous protein) and transcriptional regulatory factor ATF4 (Activating Transcription Factor 4 )were recorded after intervention with ER stress inhibitor TUDCA. The effects of er stress on valvular osteogenic transformation were analyzed.

**Result:**

After stimulation of VICs with ox-LDL, the expression levels of BMP2, PERK, CHOP, and ATF4 increased. However, TUDCA treatment can alleviate the increased expression levels of BMP2, PERK ATF4, and CHOP under ox-LDL stimulation to a certain extent.

**Conclusion:**

The endoplasmic reticulum stress signaling pathway is involved in ox-LDL-induced calcification of rabbit valve interstitial cells. Inhibition of endoplasmic reticulum stress using TUDCA can improve the progression of rabbit aortic valve calcification.

## Introduction

Calcific Aortic Valve disease (CAVD), the most common form of valvular heart disease, according to data, the global death toll of aortic valve calcification was 102700 in 2017, an increase of 101% compared with 1990 (1). Aortic valve calcification disease terminal lesions such as, aortic valve sclerosis, stenosis, incomplete closure, etc., will lead to a significant increase in cardiac load, seriously affecting people’s life and health and quality of life.

In the past, calcified valvular heart disease was often regarded as a degenerative disease of age, but in recent years, more and more studies have shown that it is an active regulation process involving multiple factors, and mechanical stimulation may be the initiating factor of this process (2), that is, early lesions of CAVD are like atherosclerosis. Changes in blood shear forces stimulate bone morphogenetic protein 2 (BMP2) and its downstream pathways and lead to injury of the valvular endothelium, followed by infiltration of lipids and inflammatory cells, the release of cytokines, calcium deposition, and valvular mineralization.

Unlike atherosclerosis, where macrophages phagocytose lipids and form foam cells, leading to calcium deposition, cardiac valve calcification involves valvular interstitial cells, resulting in osteogenic phenotype transformation and inducing calcification (3, 4).There are five main valve interstitial cells, namely embryonic endothelial/mesenchymal progenitor cells, qVICs, pVICs, aVICs and obVICs (3). AVICs play a significant role in valve calcification.

A high-fat diet can increase the serum ox-LDL level in rats, and it is often used to construct aortic valve calcification model (5), while inhibition of ER (endoplasmic reticulum) stress can protect aortic valve calcification in ApoE-/- mice fed with high-cholesterol diet (6). Therefore, we hypothesized that ox-LDL might induce calcification of valvular osteogenesis through ER stress, that is, ox-LDL is involved in osteogenesis of valvular interstitial cells through the classic ER stress pathway PERK-ATF4-CHOP, while ER stress inhibitors attenuated this effect. To this end, we isolated and cultured rabbit primary valve interstitial cells, administered ox-LDL-treated cells, observed the expression of PERK, ATF4, CHOP, and BMP2 in the ER stress pathway, and treated them with ER stress inhibitors (7). To further elucidate the role of PERK pathway in rabbit valve interstitial cell osteogenic differentiation, we hope to provide new therapeutic targets and ideas for the treatment and prevention of aortic valve calcification.

## Materials and Methods

### Animals

Eight male New Zealand white rabbits were kept in a temperature-controlled room with a cycle of 12-hour light and 12-hour darkness and fed with regular laboratory chow and unrestricted access to water. Approval of all experiments and animal care was provided by the Animal Ethics Committee of Tongji Hospital, Tongji Medical College, Huazhong University of Science and Technology.

### Extract and Culture VICs

After the aortic valve of New Zealand White Rabbits were isolated, the interstitial cells of the aortic valve were digested with trypsin containing EDTA. The culture medium was composed of Hyclone high glucose DMEM medium and Gibco 20% fetal bovine serum, supplemented with 100 U/mL penicillin and 100μg/ml streptomycin (Gibco). After 24h and 48h, the culture medium was changed respectively until the cells reached 80%-90% fusion. After the first passage, the cells were cultured with DMEM/F12 of 10% FBS. Cells from 3 to 8 generations were cultured at 37°C in a moist atmosphere with 5% carbon dioxide in the air.

### Characterization of VICs

Immunofluorescence was used to identify VICs. After the first and second antibodies of α -smooth muscle protein (α-SMA), V-WF and VemIntent were incubated successively, the cell slivers were cleaned with 4°C precooled PBS solution for 3 times, 5 min each. 0.5 mL of 1*DAPI (ABCAM) was added to stain the nuclei for 10 minutes: the cell sliders after treatment were placed under a forward fluorescence microscope for observation, and the wavelength was adjusted to 520-530nm to stimulate red fluorescence. Cell morphology was observed under a microscope of 20 and 40 times, respectively, and photographed for preservation.

### Experimental Protocols

To investigate whether ox-LDL stimulation can promote osteogenic phenotype transformation of VICs, ox-LDL (100 μg/mL) was given to stimulate aortic valve interstitial cells on day 1, day 3, and day 7, and the cells were collected to detect BMP2 mRNA levels. And BMP2 protein expression levels on day 7. The BMP2 mRNA content of aortic valve interstitial cells without ox-LDL stimulation was taken as day 0; In order to investigate whether ox-LDL stimulation can cause endoplasmic reticulum stress of VICs, VICs without ox-LDL stimulation and VICs after ox-LDL stimulation for 7 days were collected. The protein expression levels of PERK/ATF4/CHOP protein pathway in endoplasmic reticulum were determined by Western blot analysis. To investigate whether endoplasmic reticulum stress is involved in osteoblastic phenotypic transformation of aortic valve interstimal cells, the cells were divided into four groups: CTL group, ox-LDL group (100 μg/mL), ox-LDL group (100 μg/ml) +TUDCA (100umol/L, HY-19696, MCE) group and TUDCA (1mmol/L) group, in which ox-LDL (100 μg/ml) +TUDCA group cells were treated with TUDCA for 2 h, and then ox-LDL was added for 7 days. ox-LDL group cells were stimulated with ox-LDL for 7 days, and then cells in each group were collected. Western blot analysis was used to detect the expression levels of proteins related to BMP2 and ER stress signaling pathway.

### Western Blot Analysis

After being washed by ice-cold PBS, VICs were lysed in radioimmunoprecipitation assay (RIPA) lysis buffer (HY-K1001, MCE) for approximately 30 min. Then, the samples were centrifuged at 12,000 rpm at 4 ◦C for 15 min. Protein concentration was determined using the bicinchoninic acid (BCA) method (Beyotime, China). Subsequently, proteins were separated by 10% sodium dodecyl sulfate-polyacrylamide gel electrophoresis (SDS-PAGE) (EpiZyme, China) and transferred onto polyvinylidene difluoride (PVDF) membranes (IPVH00010 0.45 μm, Millipore, USA). Then, the membranes were blocked with 5% skim milk in Tris-buffered saline solution containing Tween-20 (Sigma-Aldrich, USA) for 1 h at room temperature and incubated in specific primary antibodies at 4 ◦C overnight. After detecting horseradish peroxidase-conjugated secondary antibodies for 1 h at room temperature, the proteins were visualized using enhanced chemiluminescence. The primary antibodies were as follows: Anti-CHOP antibody, Anti-BMP2 antibody, Anti-ATF4 antibody, Anti-PERK antibody, Rabbit anti-vimentin antibody, rabbit anti-vWF antibody, rabbit anti-α -SMA antibody (Wuhan Sanying Biotechnology Co., LTD).

### qRT-PCR

After the cells were treated according to the above experimental design, total RNA was extracted by Trizol method and the RNA was reversely transcribed into cDNA(reaction system: 20 dishes; Reaction conditions: 42°C2 rain, 37°C15 rain, 85°C5 S), using qRT-PCR method (reaction system: 20 anal L; Reaction conditions: “two-step reaction” was adopted, the first step was 94°C for 30 S; The second step is 94°C5 s, 60. C 30 s, 44 cycles) the mRNA levels of BMP2 and PERK were detected (see **Table 1** for primers).

**Table 1:** Primer Sequence.

Gene symbol	Forward prime sequence (5’→3’)	Reverse prime sequence (5’→3’)
BMP2	CCCAAGCTTACCACCATGGTGGCCGGGACCCGCTGTCTTC	CGCGGATCCCTAGCGACACCCACAACCCTCCAC
PERK	GCGGCAATGAGAAGTGGAAT	TCCCTCTGGGCTTAAAGGTG
GAPDH	CGCCTGGAGAAAGCTGCTA	ACGACCTGGTCCTCGGTGTA

## Result

### Cell Identification

**Figure 1:**
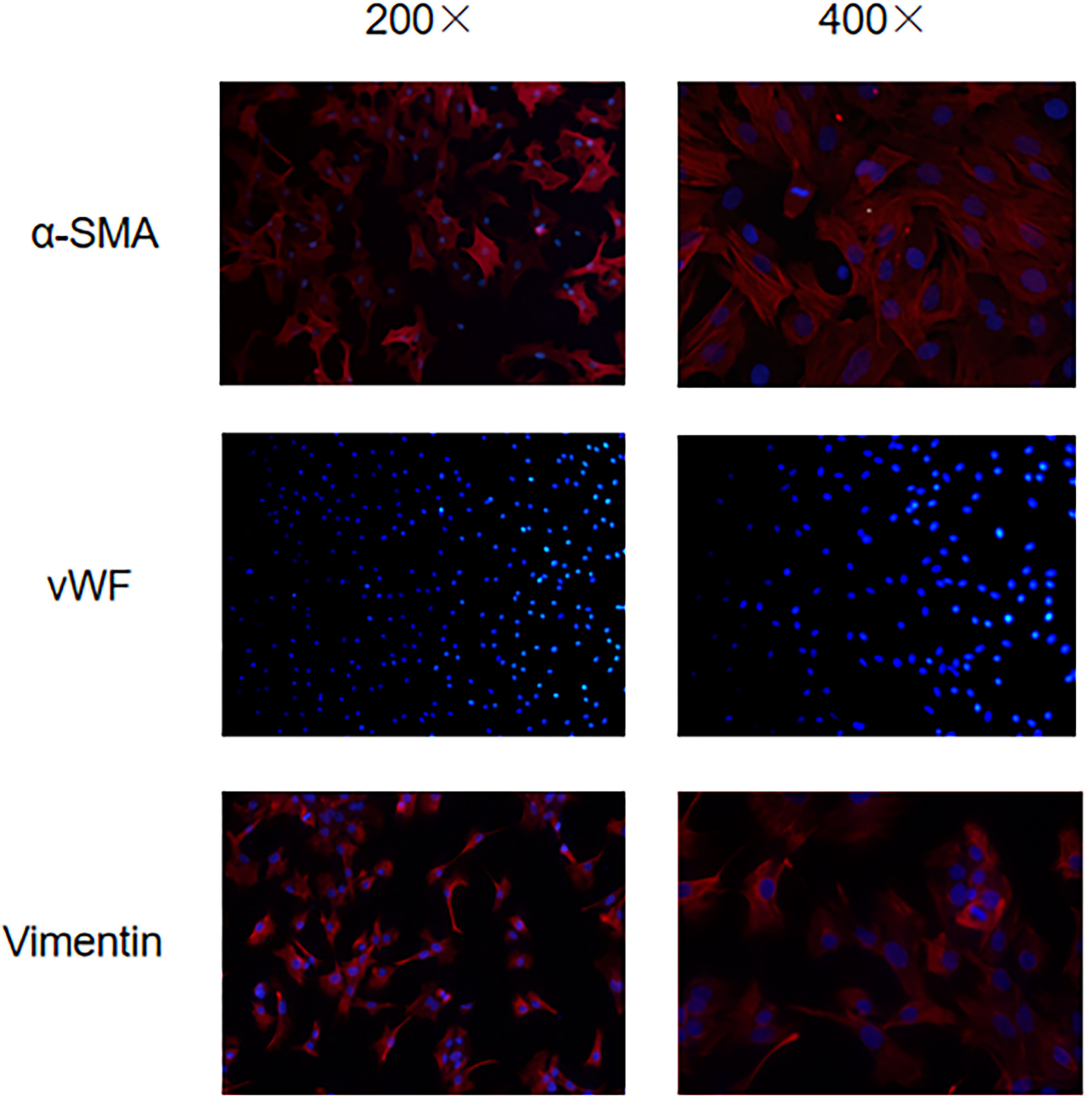
Immunofluorescence microscopy to identify the expression of α-SMA/vWF/Vimentin in VICs.

Early studies have shown that activation of aortic valve interstitial cells increases the expression of α -smooth muscle actin (α-SMA) on its surface (8). Vimentin protein is not only expressed on the valve interstitial cells, but also the valve endothelial cells, to further remove the influence of the valve endothelial cells (9), endothelial cell marker vWF is also used to identify the aortic valve interstitial cells and aortic valve endothelial cells. Therefore, α-SMA (+)/Vimentin (+)/vWF (-) cells are the de sired aortic valve interstitial cells. Under a fluorescence microscope, using green light to excite red wavelengths, the aortic valve stromal cells appear red color α-SMA (+) and Vimentin (+), indicating positive expression of α-SMA and Vimentin, vWF uses blue light to stimulate the wavelength of green light, no green fluorescence is seen, only blue stained nucleus is seen, indicating negative expression of vWF (**Figure 1**).

### ox-LDL Stimulation Can Induce Osteoblastic Phenotypic Transformation of Aortic Valve Stromal Cells

**Figure 2:**
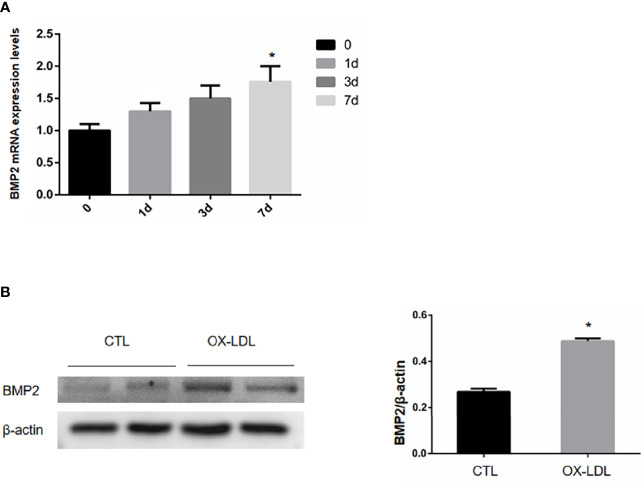
mRNA and protein expression levels of BMP2 in VIC after ox-LDL stimulation. **(A)** The relative expression of BMP2 mRNA under different time stimulation; **(B)** BMP2 protein expression in VICs after ox-LDL stimulation. * represents P value less than 0.05, indicating significant difference.

ox-LDL was given on day 1 to stimulate the aortic valve interstitial cells, and the cells were collected on day 1, 3 and 7 to detect the BMP2 mRNA content. The BMP2 mRNA content of aortic valve interstitial cells not stimulated with ox-LDL was taken as day 0, and the results showed that compared with day 0, we found that the BMP2 mRNA content increased successively on day 1, 3 and 7, and reached the peak on day 7 (**Figure 2A**). VICs was collected after ox-LDL stimulation for 7 days, and the protein expression level of osteogenic protein BMP2 was detected by WB. The results showed that the expression level of osteoblastic protein BMP2, which causes VICs, was increased after ox-LDL stimulation compared with CTL group (**Figure 2B**). These results suggest that ox-LDL stimulation can induce osteoblastic phenotypic transformation of aortic valve stromal cells.

### ox-LDL Induced Endoplasmic Reticulum Stress of VICs

**Figure 3:**
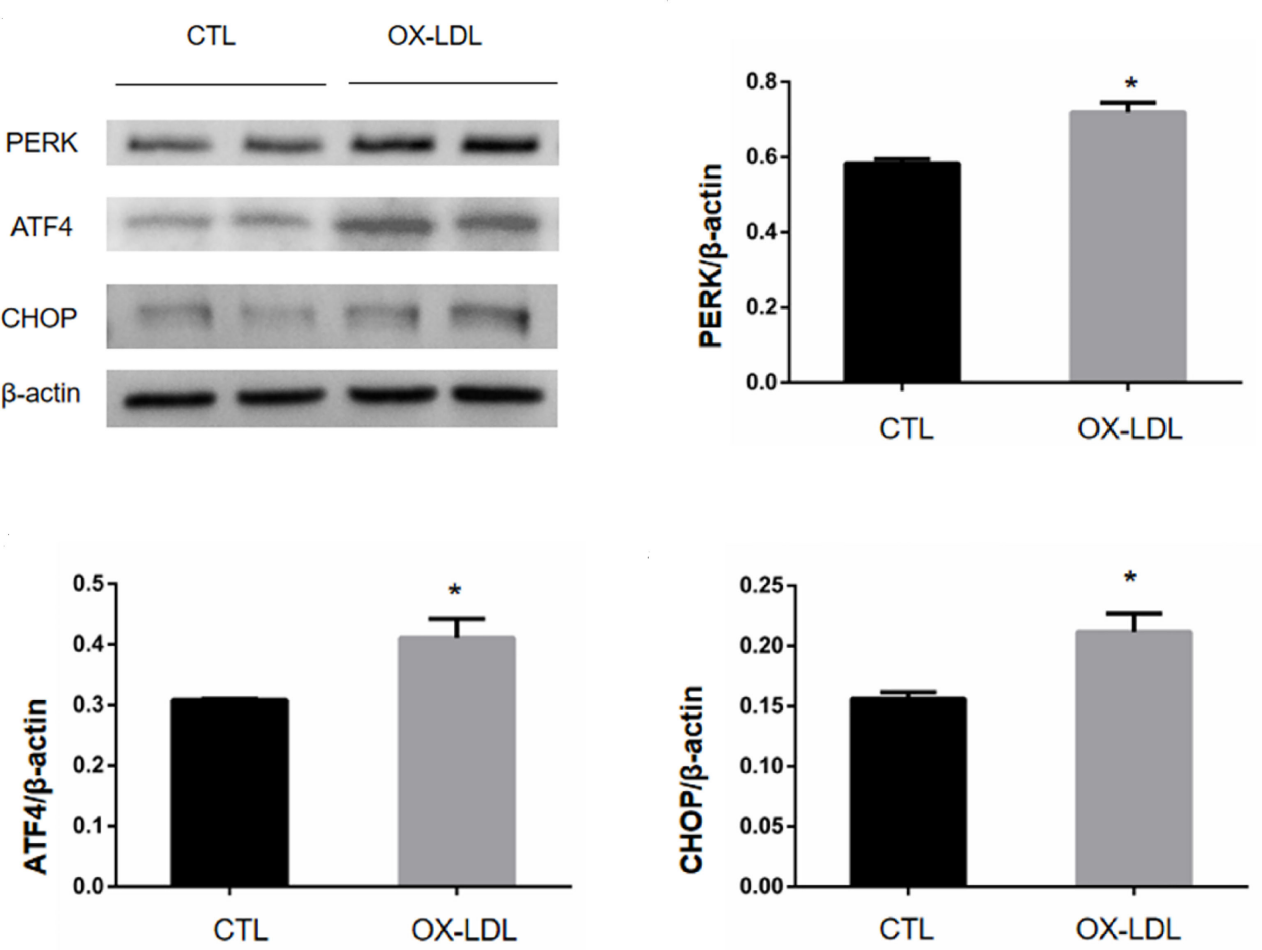
PERK/ATF4/CHOP protein expression of VICs after ox-LDL stimulation. * represents P value less than 0.05, indicating significant difference.

VICs was collected 7 days after ox-LDL stimulation, and the protein expression levels of PERK/ATF4/CHOP protein in endoplasmic reticulum were measured by WB. The results showed that compared with the CTL group, the expression levels of PERK, ATF4, and CHOP in the OX-LDL group were increased (**Figure 3**). These results suggest that ox-LDL can induce endoplasmic reticulum stress in VICs.

### Endoplasmic Reticulum Inhibitor TUDCA Can Inhibit the Osteoblastic Phenotypic Transformation Induced by ER Stress Induced by OX-LDL

**Figure 4:**
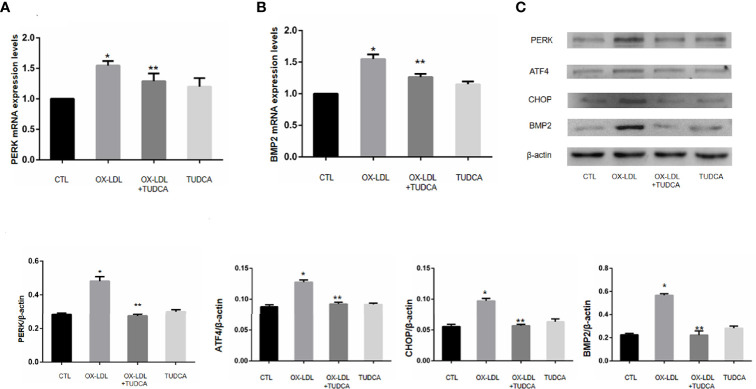
TUDCA can improve the osteogenic phenotype transformation of VICs. **(A)** PERK mRNA expression levels. **(B)**.BMP2 mRNA expression levels. **(C)** Immunoblot analysis of the pore-forming mediator of Er stress.* represents P value less than 0.05, indicating significant difference; ** means p value less than 0.01, indicating extremely significant difference.

VICs after 7 days of ox-LDL+TUDCA stimulation were collected, and the protein expression levels of PERK/ATF4/CHOP in ER stress signaling pathway were detected by WB. Compared with ox-LDL group, ox-LDL +TUDCA group reduced ox-LDL to VICs BMP2 and PERK mRNA levels to a certain extent (**Figure 4A, B**), and also alleviated ox-LDL to VICs PERK, ATF4, CHOP (**Figure 4C**), These results suggest that inhibition of ER stress may reduce osteoblastic phenotypic transformation of VICs.

## Discussion

CAVD was considered as an age-related degenerative valvular disease in the early years, but more and more evidence indicates that CAVD is an active activation process, and damage of valve endothelial cells caused by mechanical stimulation may be the cause of such diseases (2). The aortic valve is mainly composed of three lobules, each of which is primary composed of valvular endothelial cells (VECs) and valvular interstitial cells (VICs), of which the valve endothelial cells (VECs) are arranged on the outer surface of the valve, and their main role is to regulate permeability and maintain valve homeostasis, and to limit the infiltration of inflammatory cells as a barrier (10). Studies have shown that VICs increase and VECs decrease in diseased valves, and VICs are more pleomorphic than VECs (11, 12). Therefore, VICs plays an important role in the process of valve calcification, participating in valve mineralization, and ultimately leading to severe CAVD.

Therefore, we selected rabbit aortic valve, which is easy to obtain, for the experiment, and used differential centrifugation to reduce the pollution of VECs and repeated passage for purification. The cells of α-SMA (+)/Vimentin (+)/vWF (-) are the rabbit aortic valve interstitial cells (VICs) required by us (13).

ox-LDL is a common inducer of oxidative stress (14). Atherosclerosis involved in blood vessels can lead to calcification of vascular smooth muscle. However, ox-LDL can also induce ER stress and promote osteogenic effect (15). Therefore, ox-LDL was used to treat purified rabbit aortic valve cells to stimulate endoplasmic reticulum stress and osteogenesis. The results showed that ox-LDL treated VICs and its BMP2 mRNA expression increased, which was consistent with ox-LDL treated human VICs (16). It was also found that ox-LDL can also induce the activation of human VICs Notch signal, and can significantly increase the activation of inflammatory pathway NF-KB when acting together with LPS (17, 18). Therefore, it is not difficult to understand the subsequent osteogenic phenotype transformation effect of VICs caused by inflammation. However, under the action of ox-LDL, alkaline phosphatase ALP, another preosteogenic factor of VICs, did not increase significantly (19). When costimulated with LPS, the expression of ALP increased, suggesting that LPS could lead to the increase of ALP, while ox-LDL did not have such an obvious effect on ALP. ox-LDL can not only promote cell apoptosis (20, 21), but also promote the proliferation of vascular smooth muscle cells (18). However, whether ox-LDL can promote AOV calcification by increasing VIC proliferation needs further research.

Studies have confirmed that increased expression of BMP2 was found in calcified valves (22), indicating that BMP2 is involved in valve osteogenesis. We verified the osteogenic effect of ox-LDL-induced VICs at the protein level by experiments, and confirmed that the protein expression of BMP2 increased under ox-LDL stimulation. In addition, IL-37 can inhibit ox-LDL-induced valve calcification, and the increase of BMP2 expression is also inhibited (23), which also confirmed the osteogenic effect of OX-LDL-induced VICs and the regulatory effect of inflammatory factors on valvular osteogenesis.

The PERK/ATF4/CHOP pathway is the classic er pathway and the main source of CHOP protein, which is mainly involved in cell apoptosis. ox-LDL, as a common er stress inducer, can induce ER stress in cells, such as vascular endothelial cells (24) and macrophages (25). In ApoE-/- mice fed a high cholesterol diet, the deficiency of Receptor for Advanced Glycation end products (RAGE) slowed the process of valve calcification. RAGE deficiency works by inhibiting er stress. *In vitro*, HMGB1(High Molecular group Box 1 Protein) induces ER stress through RAGE to activate and promote the differentiation of AVICs osteoblasts (6). Therefore, endoplasmic reticulum stress is considered to be involved in valve osteogenesis and calcification.

Taurodeoxycholic acid (TUDCA), a hydrophilic bile acid derivative, is a classic er stress inhibitor (7). Studies have shown that TUDCA’s use is no longer limited to hepatobiliary diseases. Studies have shown that TUDCA can down-regulate the activity of PERK during ER stress in the hypothalamus of obese mice and reduce ER stress, and its neuroprotective effect has also been observed in retinal diseases (26). Thus, it has been shown to show potential therapeutic benefits in various models of many diseases, including diabetes, obesity and neurodegenerative diseases, possibly due to its cellular protective effects. The possible mechanism lies in the reduction of er stress and the stabilization of unfolded protein response. In addition, TUDCA has been found to reduce oxidative stress, inhibit apoptosis and reduce inflammation in *in vitro* and *in vivo* models of many diseases (27).

Subsequently, TUDCA was selected as an ER stress inhibitor to verify whether the use of ER stress inhibitors could improve the osteogenesis of valves. The results showed that TUDCA could inhibit er stress and osteogenesis induced by ox-LDL at protein and gene levels. Studies have shown that the expression levels of VICs phosphorylated IRE-1,PERK,eIF2α, ATF4 and RUNX2 in CAVD patients were down-regulated to varying degrees after the addition of TUDCA (28), which confirmed that ER stress is involved in valvular osteogenesis, and er stress inhibitors can inhibit ER stress and valvular osteogenesis. Therefore, combined with the experimental results, we concluded that ox-LDL-mediated ER stress is involved in regulating the osteogenic phenotype transformation of rabbit aortic valve interstitial cells.

In this study, the gene and protein expression levels of BMP2, PERK, ATF4, and CHOP in ox-LDL-stimulated valvular interstitial cells showed differences between the experimental group and the control group, and the transcription factor ATF4 downstream of PERK was confirmed to be involved in osteogenesis (29). It can be speculated that ATF4 in the PERK pathway has a significant correlation with BMP2 expression, but the specific mechanism in valve interstitial cells remains to be further studied.

This study verified that ox-LDL-induced ER stress is involved in the phenotypic transformation of VICs, and inhibition of ER stress can reduce the osteogenic effect of VICs. However, the relationship between other ER stress pathways and CAVD, such as IRE1, ATF6 pathway and changes of related transcription factors and proteins, needs further verification. Although this study is only a small finding, it is expected to provide some ideas for future treatment of CAVD.

## Data Availability Statement

The raw data supporting the conclusions of this article will be made available by the authors, without undue reservation.

## Ethics Statement

The animal study was reviewed and approved by the Tongji Hospital Committee for Ethical Approval for Research Involving Animals.

## Author Contributions

YT was the guarantor of the article, YG and YL drafted the manuscript, WD, XX, HZ, and LZ reviewed the manuscript. All authors contributed to the article and approved the submitted version.

## Funding

This study is supported by the National Science Foundation of China (no. 81070190) And Hubei Chen Xiaoping Science and Technology Development Fund (CXPJJH12000005-07-40).

## Conflict of Interest

The authors declare that the research was conducted in the absence of any commercial or financial relationships that could be construed as a potential conflict of interest.

## Publisher’s Note

All claims expressed in this article are solely those of the authors and do not necessarily represent those of their affiliated organizations, or those of the publisher, the editors and the reviewers. Any product that may be evaluated in this article, or claim that may be made by its manufacturer, is not guaranteed or endorsed by the publisher.
